# Study of Molybdenite Floatability: Effect of Clays and Seawater

**DOI:** 10.3390/ma15031136

**Published:** 2022-02-01

**Authors:** Catalina Soto, Norman Toro, Sandra Gallegos, Edelmira Gálvez, Aurora Robledo-Cabrera, Ricardo I. Jeldres, Matías Jeldres, Pedro Robles, Alejandro López-Valdivieso

**Affiliations:** 1 Department of Metallurgical and Mining Engineering, North Catholic University, Antofagasta 1270709, Chile; catsotomadrid@gmail.com (C.S.); egalvez@ucn.cl (E.G.); 2 Faculty of Engineering and Architecture, Arturo Prat University, Iquique 1100000, Chile; notoro@unap.cl (N.T.); chichined@gmail.com (S.G.); 3 Institute of Metallurgy, Autonomous University of San Luis Potosi, Av. Sierra Leona 550, San Luis Potosí 78210, Mexico; acabrera@uaslp.mx; 4 Departamento de Ingeniería Química y Procesos de Minerales, Facultad de Ingeniería, Universidad de Antofagasta, Av. Angamos 601, Antofagasta 1240000, Chile; hugo.jeldres.valenzuela@ua.cl; 5 Escuela de Ingeniería Química, Pontificia Universidad Católica de Valparaíso, Valparaíso 2340000, Chile; pedro.robles@pucv.cl

**Keywords:** seawater flotation, molybdenite, kaolin, Na-montmorillonite

## Abstract

Current challenges in froth flotation are the presence of complex gangues and the use of low-quality waters, such as seawater. In this scenario, the recovery of molybdenum minerals is difficult, mainly due to the hydrophobic faces’ physicochemical changes. In the present study, the natural floatability of pure molybdenite was analyzed by using microflotation assays, and hydrophobicity was measured by performing contact-angle measurements. The impact of two clays, kaolin (non-swelling) and Na-montmorillonite (swelling), was studied. The behavior in freshwater and seawater at pH 8 was compared, considering the current condition of the Cu/Mo mining industries, which use seawater in their operations. The presence of clays lowered the natural floatability of molybdenite precisely because they adhere to the surface and reduce its contact angle. However, the intensity with which they cause this phenomenon depends on the type of water and clay. Kaolin strongly adheres to the valuable mineral in both freshwater and seawater. For its part, Na-montmorillonite does it with greater intensity in a saline medium, but in freshwater, a high concentration of phyllosilicate is required to reduce the hydrophobicity of molybdenite. The clays’ adherence was validated by scanning electron microscopy (SEM) analysis.

## 1. Introduction

Molybdenite (MoS_2_) is the most important primary source of molybdenum, which is essential for its several physical properties, such as stability and resistance to high temperatures, high thermal and electrical conductivity, resistance to attack by molten metal, and high rigidity. Molybdenum and its alloys are used in lighting, electrical and electronic devices, medical equipment, high-temperature furnaces, and thermal-spray coating [1,2].

Molybdenite can be recovered by froth flotation supported by its hydrophobic character. It has a hexagonal structure consisting of a single sheet of molybdenum atoms sandwiched between two sheets of sulfur atoms. It exhibits two structures on its surface: the faces, which are formed by the breaking of the van der Waals sulfur–sulfur bonds; and the edges, which are generated by breaking a strong covalent molybdenum–sulfur bond, called charged edges [3,4,5]. As a result, the edges are hydrophilic and the faces hydrophobic, with the latter being responsible for providing natural floatability to the mineral [6]. In contrast to other sulfides, molybdenite does not require collectors, although it is common to use non-polar oils, which improve recovery [7,8].

The shape and size of the particles are of great importance [9], since, the higher the face/edge aspect ratio, the greater the chance of appearing in the concentrate. Molybdenite fines have a lower aspect ratio (compared to coarser particles), so they exhibit low floatability and kinetics rates. For example, López-Valdivieso et al. [10] showed that, at neutral pH, molybdenite presents a recovery of less than 20% at a size range of 38–45 µm, while, for a size between 75 and 150 µm, the recovery increased to 60%.

Generally, molybdenite concentrate is a by-product of the selective flotation of Cu-Mo minerals. After a first rougher flotation stage, molybdenum minerals are collected in the concentrate, while copper sulfides are depressed by adding sodium hydrosulfide (NaHS) [11]. These stages involve a large consumption of water, generating significant environmental impacts, mainly in those localities located in arid zones, such as in the north of Chile, the south of Peru, and Australia [12]; this forces us to find alternatives that face the scarcity of water resources. The use of seawater has become very important in mining processes in recent years, and there are even companies that use direct seawater without a desalination process [11,13,14]. However, salinity is not an impediment to the floatability of molybdenite at neutral pH conditions. In fact, Lucay et al. [13] suggested that the recovery of molybdenite fine particles could be improved in saline solutions. The authors explained their results with the DLVO theory, based on the reduction of electrostatic repulsion between the bubbles and the anionic edges of the molybdenite. However, it is common for copper-moly ores to be processed under highly alkaline conditions, avoiding pyrite recovery [15,16]. This strategy generates good results in freshwater, considering that molybdenite does not lose its floatability when the pH is raised. Lopez-Valdivieso et al. [10] analyzed the hydrophobicity of the molybdenite surface over a wide pH range (pH 5–12), finding that there are no significant alterations of the contact angle in distilled water. However, this method cannot be implemented in seawater, where the formation of solid calcium and magnesium precipitates adsorb on the surface of molybdenite, forming a hydrophilic coating that impairs its natural hydrophobicity [6]. Therefore, the approach adopted is to work at the natural pH (close to pH 8), applying new reagents that allow pyrite to be depressed [17].

Additionally, the presence of clays is a recurring challenge in the copper industry and is a constant subject of research, considering that it affects practically all stages of mineral processing [18,19].

Clays are phyllosilicates whose structure is composed of fine- and ultrafine-grained minerals. They have two crystallographically different surfaces, namely the faces that tend to be negatively charged and the edges that vary their charge according to the pH [20]. These particles may coagulate with valuable minerals, generating a hydrophilic layer around the surface that impairs contact with the collector and bubbles (coating effect) [21]. It has also been mentioned that clays can consume reagents, such as collectors, which lower their selectivity [22].

Among the most common clays are species from the kaolin group (e.g., kaolinite) and the smectite group (e.g., Na-montmorillonite). Kaolinite and Na-montmorillonite are finely divided crystalline aluminosilicates of colloidal sizes with two-dimensional arrays of silicon–oxygen tetrahedral sheets and octahedral alumina or magnesium–oxygen sheets. In kaolinite, one silica sheet and one alumina sheet (TO; 1:1) share oxygen atoms, resulting in a two-layer mineral. In Na-montmorillonite, an octahedral alumina sheet shares oxygen atoms with two silica sheets (TOT; 2:1), resulting in a three-layer mineral. These clays have anionic-electric-charge sites on the basal planes, due to substituting the Si and Al in the crystal lattice for cations of lower positive valence. Exchangeable cations compensate for this excess of negative lattice charge. The polar tetrahedral SiOH and octahedral AlOH sites on the edges of the clays interact with H^+^ and OH^−^, giving rise to positive or negative charges, depending on the pH. This surface-charge heterogeneity of the clays governs the interaction of clays with other minerals in slurries.

Furthermore, Na-montmorillonite is highly swellable, whereas kaolinite does not alter its volume in an aqueous solution. The swelling of Na-montmorillonite is less in saline waters than in freshwaters, because there is a higher concentration of cations in the interlaminar space, which reduces the electrostatic repulsion between them and, therefore, shortens their separation distance. This limits the number of water molecules that can enter the interlaminar space of the phyllosilicate [23].

The effect of clays on copper flotation has been extensively studied, including systems in different water qualities [18,24,25]. However, systematic work on molybdenum floatability is scarce. Some recent research stands out, such as the work of Yepsen et al. [26]. The authors evaluated the effect of muscovite and biotite on the flotation of chalcopyrite and molybdenite in seawater. Both phyllosilicates reduced the recovery and grade of the concentrate of an artificially prepared mineral. Ramírez et al. [25] analyzed the impact of kaolinite on the flotation of molybdenum in seawater. The results showed a depressant effect throughout the analyzed pH range (pH 7–11); however, it intensifies at pH> 9, when the formation of solid calcium–magnesium complexes begins. The authors alleviated the depression with sodium hexametaphosphate, which increased the repulsive forces between molybdenite and precipitates.

This study deepens the current knowledge on the impact that different clays generate on the floatability of molybdenum minerals in seawater. Clays of the kaolin group of a non-swelling nature and Na-montmorillonite of a swelling character were evaluated. The changes in floatability generated according to the type of water and clay were related to the degree of hydrophobicity of the molybdenite face obtained through contact-angle measurements. The clays’ adherence was analyzed by using scanning electron microscopy (SEM).

## 2. Methodology

### 2.1. Materials

**Figure 1:**
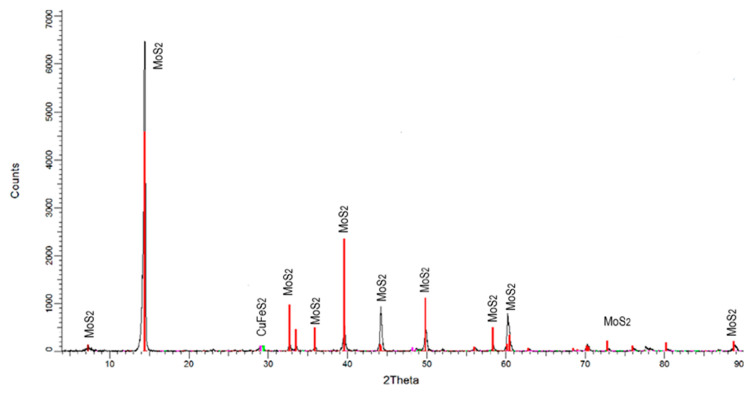
X-ray diffraction of molybdenite samples.

**Figure 2:**
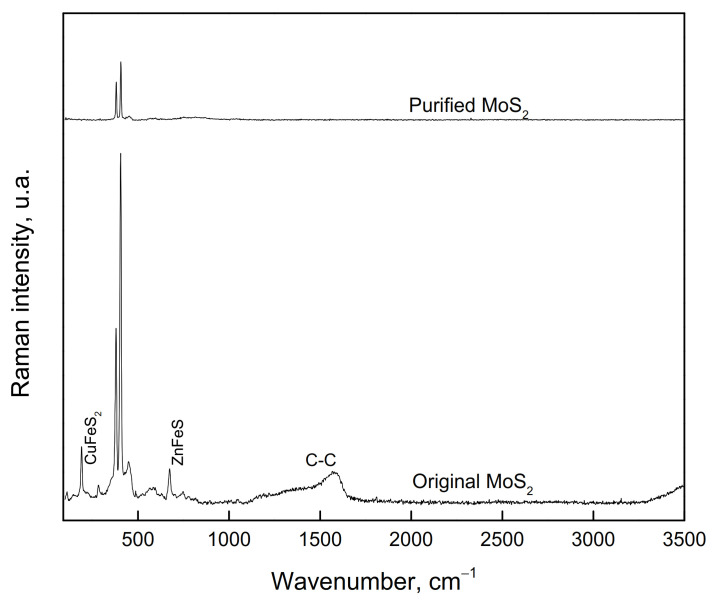
Raman spectrum of molybdenite samples before and after purification with acetone.

For the microflotation tests, a MoS_2_ concentrate from a Cu-Mo secondary mineral concentrator plant, Sonora, Mexico, was used. This sample was cleaned to remove Cu, Fe, and silicate mineral impurities by floating the MoS_2_ in deionized water, using only the MIBC frother. This flotation process was carried out five times. The recovered MoS_2_ concentrate was filtered and dried. The size fraction −150 + 75 µm was obtained from this concentrate for the microflotation tests. Before being used, the sample was washed with acetone to remove the organics present on the particle surfaces. The washing was repeated several times until organic species were no longer detected by Raman spectrometry (Thermo Scientific DXR Raman Microscope by Thermo Fisher Scientific Inc, Madison WI, USA). The sample was also characterized by using X-ray diffraction (XRD, Brucker D8 Advance by Brucker AXS GmbH, Karlsruhe, Germany). It was also chemically analyzed for purity. Figure 1 and Figure 2 show the XRD spectrum (CuK-alpha) and Raman spectrum of molybdenite before and after washing with acetone, respectively. The XRD spectrum reveals that the sample consists mainly of MoS_2_ with small amounts of chalcopyrite. The chemical analysis by Mo, S, Fe, and Cu determined that the purified sample contains 94.7% MoS_2_, 3.3% CuFeS_2_, and 3.0% FeS_2_. The Raman spectrum revealed that molybdenite does not contain organic compounds on its surface, and this could interfere with its floatability.

Molybdenite crystals 3/4 “long and 1/3” wide were used for contact-angle measurements. Na-Montmorillonite from Sonora, Mexico, was used. Kaolin samples from Sigma Aldrich (Taufkirchen, Germany) was used in this work. For all flotation tests and contact-angle measurements, deionized water and synthetic seawater with the salt composition indicated in Table 1 were used.

### 2.2. Microflotation

Microflotation tests were carried out in a glass Hallimond tube, manufactured by a glassware (Puebla, México). The tests were carried out using 1 g of MoS_2_ and 120 mL of deionized water and synthetic seawater. Without clays, the mineral was conditioned for 5 min at pH 8 to transfer the suspension to the Hallimond tube. The effect of clays on the natural floatability of MoS_2_ was determined by conditioning the mineral with variable amounts of clay for 5 min at pH 8. MoS_2_ flotation was for 1 min, using pure N_2_ at a 10 mL/min flow. The floatable products and the tails were collected, filtered, dried, and weighed to determine the recovery of MoS_2_.

### 2.3. Contact Angle

A Ramé-Hart model 100-00 115 NRL CA equipment with its DROPImage Standard program, manufactured by Ramé-Hart Instrument Co. Saccasunna NJ, USA, was used for the contact-angle measurements. The MoS_2_ sample was placed inside a 300 mL quartz cell in the absence and presence of the desired clays. The contact time of the clay with the MoS_2_ crystal was 40 min. An air bubble formed by a J-shaped plastic tube inserted into an Omnican syringe was placed on the face of the MoS_2_ crystal. At least five contact-angle measurements were made in deionized water and synthetic seawater. In this work, the average value is reported.

## 3. Results and Discussion

### 3.1. Kaolin Effect on MoS_2_-Face Contact Angle and MoS_2_ Floatability

**Figure 3:**
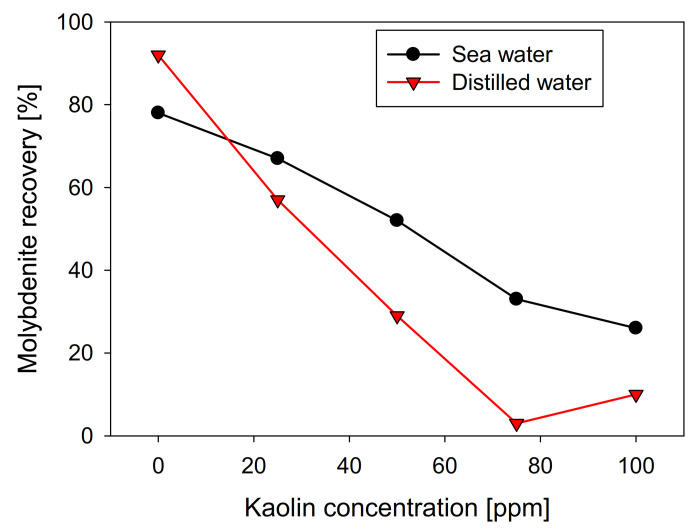
Effect of kaolin concentration on recovery of molybdenite, using distilled water and synthetic seawater.

Figure 3 shows the results obtained for microflotation of molybdenite in the presence of kaolin, considering freshwater and synthetic seawater. In the absence of clays, the floatability of molybdenite was higher with distilled water, where 92% was recovered while, in seawater, the recovery was 79%. It should be noted that Qiu et al. [25] obtained a contrary trend, with recoveries close to 85% in distilled water, rising to 95% in seawater, at pH 8. However, the researchers developed their experiments by using a classic collector of the copper industry, potassium amyl xanthate (PAX), but in our study, only a frother agent (MIBC) was applied. Another relevant difference is the particle size. Qiu et al. [25] used particles in the range of 38–75 µm, while, in our work, the particles were 75–150 µm. This could explain the need for Qiu et al. [25] to use a collector, since finer particles increase the edge/face ratio, leading to greater hydrophilicity and a greater energy barrier between the particles and bubbles. In this context, it is expected that the effect of salinity strongly depends on the particle size. In a range of fine particles, the high ionic charge may benefit the flotation performance, whereas, in coarse particles, which are more hydrophobic, seawater affects performance.

**Figure 4:**
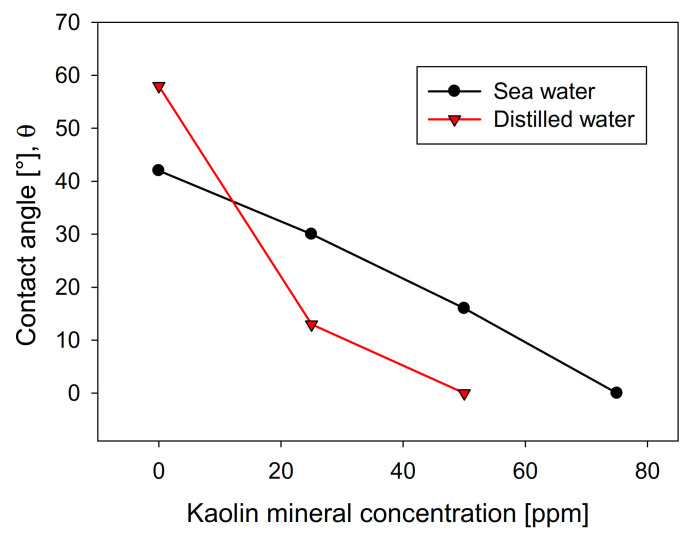
Effect of kaolin concentration on molybdenite surface contact angle, using distilled water and synthetic seawater.

The recovery shows a direct relationship with the contact angle of the molybdenite surface, since, in distilled water, it has a value greater than 58°. In comparison, in seawater, it is slightly less than 42°. However, the presence of kaolinite reduces the floatability of sulfide in both types of water, generating a more abrupt decay in distilled water, where a floatability of less than 10% was obtained at a concentration of 75 ppm of kaolinite. In comparison, 35% was obtained in synthetic seawater with the same phyllosilicate. The same trend is observed in the contact angle. Figure 4 shows that the molybdenite face completely loses its hydrophobicity (contact angle = 0°) with 50 ppm of clay, while a concentration close to 80 ppm was required in seawater. These results can be explained in terms of the DLVO theory, since the kaolinite, which is the main constituent of kaolin, would present neutral/cationic edges that can bind to the surface of the molybdenite, especially in its anionic edges and micro-edges, which are negatively charged even at acidic pH [26,27]. On the other hand, the ions present in seawater compress the electrical double layer around the particles, where the magnitude of the negative zeta potential of kaolin went down from −39 mV in distilled water to −5 mV in seawater. For montmorillonite, it went down from −35 to −9 mV. This leads to a heterocoagulation in seawater between the clays and the valuable mineral. This phenomenon is similar to that reported by Yepsen et al. [26], who studied micas’ effect on the floatability of molybdenite in seawater, and Ramírez et al. [27], who analyzed the impact of kaolinite on molybdenite flotation in seawater. This latter used sodium hexametaphosphate to induce dispersion between the minerals. The authors achieved promising results at the lab scale.

**Figure 5:**
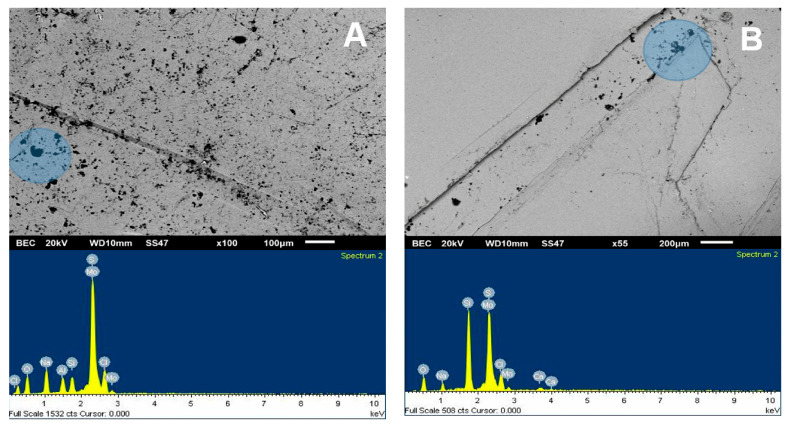
SEM image (**upper**) and chemical spectrum of the analysis zone of molybdenite crystal (**lower**) coated by kaolin at 75 ppm: (**A**) deionized water and (**B**) seawater.

**Figure 6:**
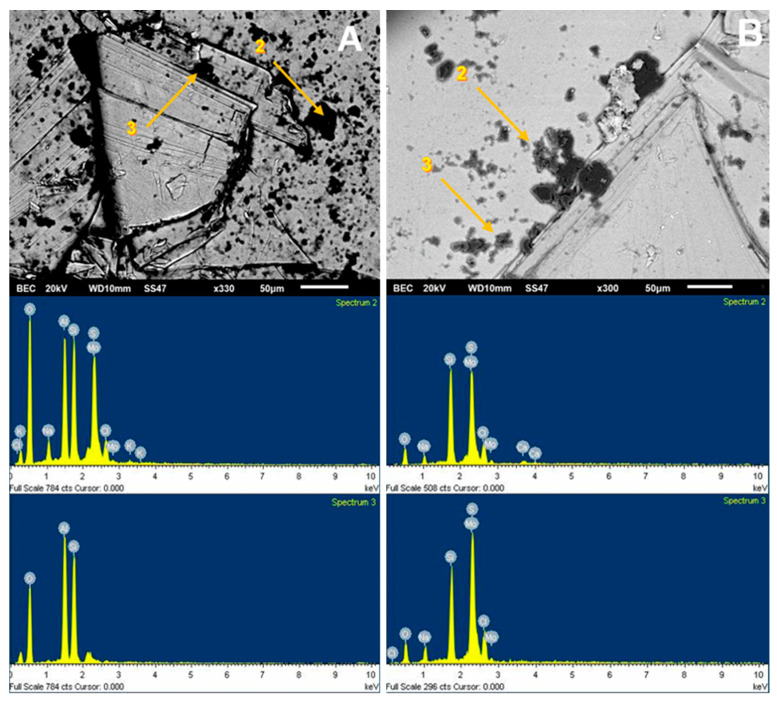
SEM image (**upper**) and chemical spectrum of the molybdenite analysis zone (**lower**), selected in the left zone of the mineral and coated by kaolin at 75 ppm: (**A**) deionized water and (**B**) seawater.

The SEM analysis presented in Figure 5 was used to visualize the adhesion of kaolin particles on the molybdenite surface, both in distilled water and seawater. For this, a concentration of 75 ppm of kaolin was used. Dark crystal-shaped particles, with an average size of less than 10 µm, were seen spreading across the entire surface of the valuable mineral when it was immersed in deionized water (Figure 5A). Supported by the chemical spectrum of the analysis area, it is observed that the matrix has abundant clay particles, with the presence of chlorine, sodium, aluminum, silicon, and oxygen. The blue shaded area is a 1900× zoom that is shown in Figure 6A. When analyzing two random zones of the glass, we found zones with aluminum, silica, oxygen, sodium, and chlorine.

Figure 5B shows the images of molybdenite exposed to a synthetic seawater environment in the presence of kaolin. Although clays adhered to the surface of the molybdenite, these were not found in the same proportions as in the case with deionized water, thus clearly highlighting that the attraction between both minerals is weakened in a saline medium. On the other hand, the chemical analysis of the species in the crystal matrix (mainly composed of silica and oxygen) shows low chlorine and sodium ion levels. The other ions, such as calcium, potassium, aluminum, and magnesium, were low but detectable.

Interestingly, the SEM images are consistent with the floatability (Figure 3) and contact-angle (Figure 4) tests. The greater the affinity of kaolin with the surface of molybdenite, the more significant the hydrophilicity is, and, consequently, the floatability is reduced.

### 3.2. Na-Montmorillonite Effect on MoS_2_-Face Contact Angle and MoS_2_ Floatability

**Figure 7:**
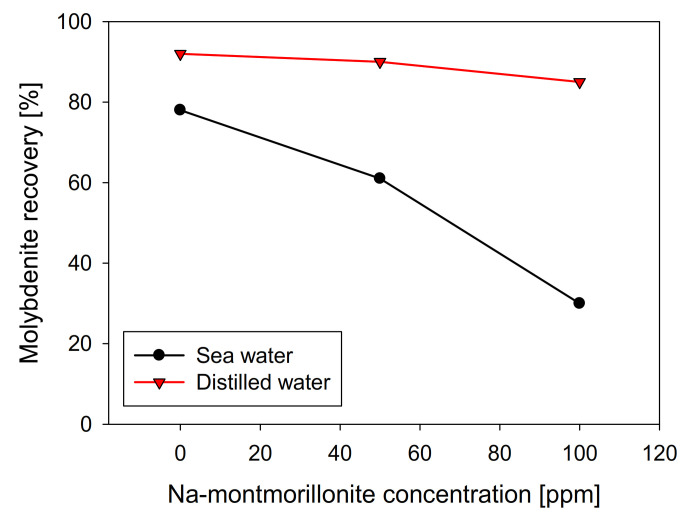
Recovery of molybdenite with respect to Na-montmorillonite concentration, using distilled water and synthetic seawater.

Na-montmorillonite has a similar effect to kaolin when seawater was used, presenting a practically linear recovery decrease with respect to the clay concentration, obtaining a value close to 30% in the presence of 100 ppm of Na-montmorillonite. However, in distilled water, its behavior differs significantly, and as can be seen in Figure 7, molybdenite practically does not see its recovery affected, obtaining around 85% with 100 ppm of Na-montmorillonite. Curiously, under these conditions, in the presence of kaolin, the recovery of molybdenite was less than 10% (Figure 3).

**Figure 8:**
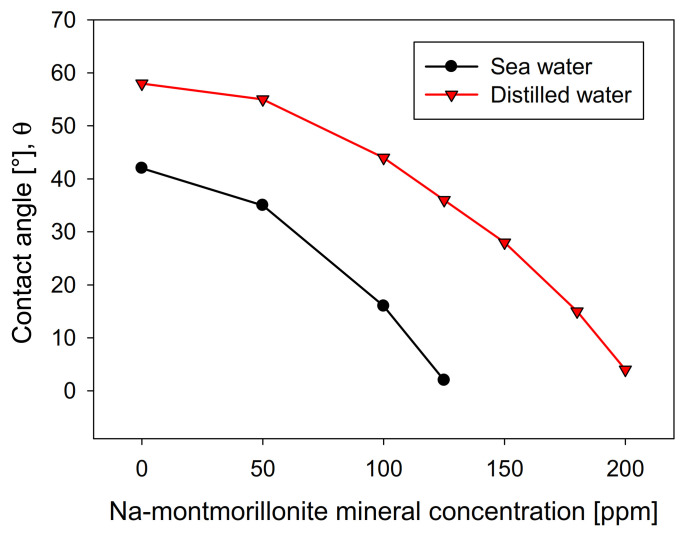
Effect of Na-montmorillonite concentration on molybdenite surface contact angle, using distilled water and synthetic seawater.

Figure 8 shows the influence of the Na-Montmorillonite concentration on the contact angle of the bubbles on the crystal surface, using the two types of waters studied. The curves show a similar trend in both cases, although distilled water is above seawater in all concentration ranges. This is contrary to kaolin (Figure 4), wherein, above 10 ppm, the contact angle presented significantly lower values in distilled water than in seawater. The greater the presence of Na-montmorillonite, the more the contact angle of the bubbles decreases, until it is entirely hydrophilic, a situation that occurs at a clay concentration much higher than that seen with kaolin. This difference is especially notable in distilled water, where the amount required to achieve a contact angle close to zero is approximately four times greater than kaolin. Interestingly, under these conditions, Na-montmorillonite has the most significant swelling effect [28].

It should be mentioned that, in a microcell, the rheological conditions are negligible, considering that the solid concentration is approximately 1%. This implies that the recovery is exclusively associated with the hydrophobic character, showing that the swelling of montmorillonite does not significantly affect the floatability. However, it is not ruled out that these results are not replicated in a larger cell, considering that the rheological characteristics are accentuated.

**Figure 9:**
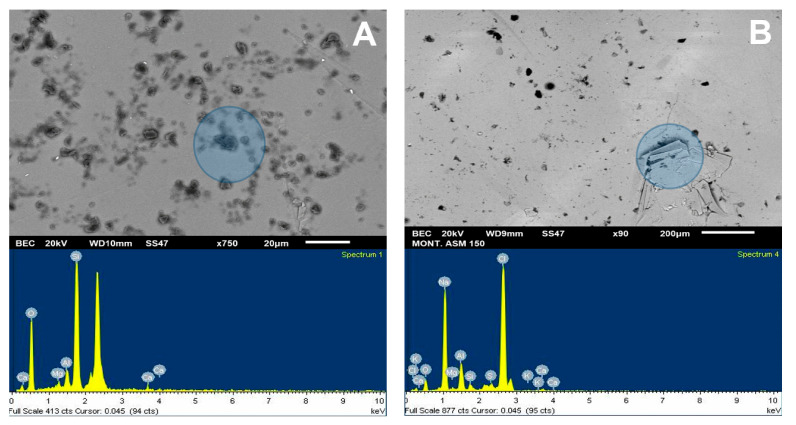
SEM image (**upper**) and chemical spectrum of the analysis zone of molybdenite crystal (**lower**) coated by Na-montmorillonite at 200 ppm: (**A**) deionized water and (**B**) seawater.

**Figure 10:**
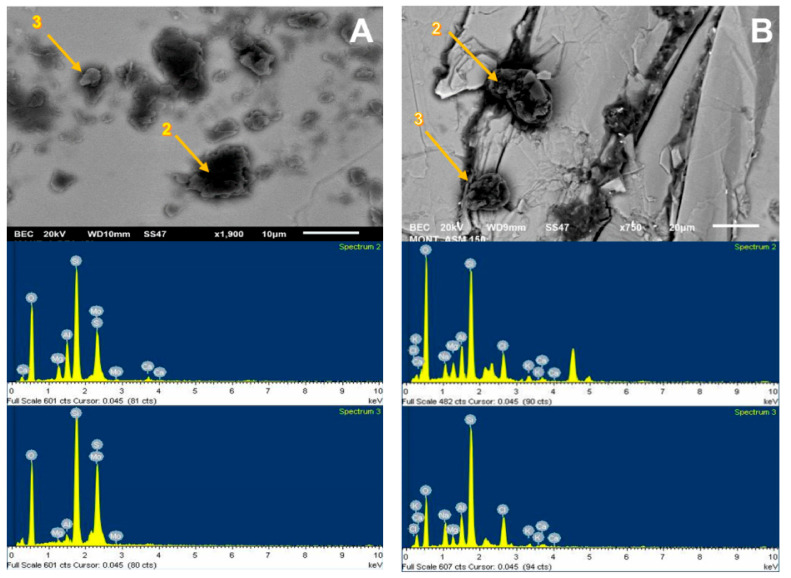
SEM image (**upper**) and chemical spectrum of the molybdenite analysis zone (**lower**), selected in the left zone of the mineral, coated by Na-montmorillonite at 200 ppm: (**A**) deionized water and (**B**) seawater.

For the SEM analysis of the molybdenite surface, 200 ppm of Na-montmorillonite was considered, since, in this condition, the surface of the sulfide is hydrophilic. SEM analysis, as presented in Figure 9A, was used to visualize and verify that the clay particles adhere to the surface. Dark particles in the form of crystals are seen in all areas of the mineral, with an average size of less than 10 µm, supported by the chemical spectrum in the analysis sector. The most prominent peaks correspond to Si and Mo, respectively, with calcium, magnesium, aluminum, and oxygen ions. The same figure also shows a blue shaded area where a 1900× zoom was applied, taking new samplings to obtain the results of Figure 10A. The particles correspond to silica and oxygen structures smaller than 10 µm, and the darker crystals formed to have more calcium cations.

Figure 9B shows the SEM results of the analysis of the molybdenite crystal in the presence of Na-Montmorillonite of 150 ppm and synthetic seawater. The chemical spectrum of the analyzed area shows a high presence of chlorine and sodium ion, followed by potassium, calcium, magnesium, silica, and oxygen. The increased presence of Na and Cl suggests that NaCl crystallized and precipitated on the surface of the mineral. It should be noted that the chemical spectrum detects chemical elements but not how they are grouped. The same figure also shows a blue shaded area where a 750× zoom was applied to the section, taking new samples at the site; the results are shown in Figure 10B. Three out of four chemical analyses can be seen in the area: (1) it is found that the molybdenite matrix does not have significant amounts of other ions; (2) and (3) crystal on the surface in which high SiO contents are detected, along with other cations of interest; and (4) high contents of chlorine and sodium are seen.

It is possible to detect the clear influence of the type of water used on the crystal structure adsorbed on the molybdenite surface. In seawater, they are larger than 20 µm and are better formed, while in deionized water, they have a more amorphous structure and are smaller than 10 µm.

This study clearly demonstrated that the clays Na-montmorillonite and kaolinite are detrimental to molybdenite flotation, particularly when using seawater. These clays are common in porphyry copper-molybdenite ores. Therefore, the recovery of molybdenite would lower due to the adhesion of these clays on the hydrophobic face of molybdenite. Two ways can prove this: (1) desliming the milled ore to remove the clays prior to the flotation step and (2) using clays dispersants to prevent the attachment of the clays on molybdenite.

## 4. Conclusions

The floatability of pure molybdenite minerals was analyzed, evaluating the impact of two types of clays, kaolin (non-swelling) and Na-montmorillonite (swelling). The behavior in freshwater and seawater was compared, and the results were linked to the changes in the hydrophobicity of the mineral through contact-angle measurements and the number of clay particles that can be adsorbed to the valuable mineral, using SEM image analysis and the chemical spectrum of the surface.

Both clays adhere to the molybdenite, reducing its floatability. However, the intensity at which the phyllosilicates affect the process depends on the type of water. In distilled water, a high density of kaolin particles appeared on the molybdenite surface. It is postulated that the main adsorption mechanism is through electrostatic attraction, since the kaolinite edges are neutral/cationic and the molybdenite edges are anionic at the pH conditions in which the experiments were carried out. However, the presence of ions in seawater mitigated the attractive forces between both minerals, which implied an increase in the contact angle and floatability of the valuable mineral. Na-montmorillonite, an expandable clay in freshwater, is adsorbed lower than kaolin. This generated a less noticeable change in the contact angle and floatability of the sulfide. However, the detrimental effect of this phyllosilicate was much more intense in seawater. This behavior coincides with the reduction of the swelling effect of Na-montmorillonite, which could facilitate its adsorption on the molybdenite surface.

## Figures and Tables

**Table 1:** Chemical composition of synthetic seawater used for the experiment in one liter of solution.

Synthetic Seawater (SSW)	
Salt	Mass (g)
Na_3_PO_4_ · 12H_2_O	0.0046
NaHCO_3_	0.2100
KCl	0.7753
CaCl_2_	2.3639
MgCl_2_ · 6H_2_O	2.6907
Na_2_SO_4_	4.1476
NaCl	23.6098

## Data Availability

The data presented in this study are available upon request from the corresponding author.
